# Effects of tDCS on Tactile Perception Depend on Tactile Expertise in Both Musicians and Non-Musicians

**DOI:** 10.3390/brainsci10110843

**Published:** 2020-11-12

**Authors:** Ben Godde, Lev Dadashev, Ahmed A. Karim

**Affiliations:** 1 Department of Psychology and Methods, Jacobs University, 28795 Bremen, Germany; carpelev@gmail.com (L.D.); ahmed.karim@uni-tuebingen.de (A.A.K.); 2 University Clinic of Psychiatry and Psychotherapy, University of Tübingen, 72074 Tübingen, Germany

**Keywords:** tDCS, expertise, tactile, detection, discrimination

## Abstract

Brain plasticity in the somatosensory cortex and tactile performance can be facilitated by brain stimulation. Here, we investigated the effects of transcranial direct current stimulation (tDCS) on tactile perception in musicians and non-musicians to elucidate how tDCS-effects might depend on tactile expertise. On three separate days, 17 semi-professional musicians (e.g., piano or violin players) and 16 non-musicians aged 18–27 years received 15 min of 1 mA anodal (a-tDCS), cathodal (c-tDCS) or sham tDCS in a pseudorandomized design. Pre and post tDCS, tactile sensitivity (Touch Detection Task; TDT) and discrimination performance (Grating Orientation Task; GOT) were assessed. For further analysis, the weekly hours of instrument-playing and computer-typing were combined into a “tactile experience” variable. For GOT, but not TDT, a significant group effect at baseline was revealed with musicians performing better than non-musicians. TDT thresholds were significantly reduced after a-tDCS but not c-tDCS or sham stimulation. While both musicians’ and non-musicians’ performance improved after anodal stimulation, neither musical nor tactile expertise was directly associated with the magnitude of this improvement. Low performers in TDT with high tactile experience profited most from a-tDCS. We conclude that tactile expertise may facilitate somatosensory cortical plasticity and tactile learning in low performers.

## 1. Introduction

A causal link between tactile stimulation, tactile perception, and brain plasticity has been widely accepted (for review, cf. [1]). Various studies demonstrated, for example, that peripheral tactile stimulation using Hebbian coactivation within or across fingers induced somatosensory cortical plasticity and tactile learning already after short-term exposure [2,3,4]. Additionally, by using functional magnet resonance imaging (fMRI), Hodzic and colleagues [5] revealed that peripheral tactile stimulation induced cortical reorganization, namely in the primary (S1) and secondary (S2) somatosensory cortices and that the magnitude of changes was positively correlated with the amount of improvement in a tactile Grating Orientation Task (GOT).

Besides peripheral tactile stimulation, also direct brain stimulation has demonstrated positive results in tactile detection and discrimination. Such brain stimulation can be applied as either an online or offline stimulation protocol. In online stimulation protocols, cortical excitability and behavioral outcomes are measured during stimulation. Online stimulation can also be applied at the same time as a training or learning intervention to enhance plasticity and learning. In this study, we used offline brain stimulation. With offline stimulation, it is assumed that the effects of brain stimulation persist for some time (minutes to hours) and behavioral correlates of altered neural mechanisms are measured before and after brain stimulation. The first lot of studies in this field employed Transcranial Magnetic Stimulation (TMS). For example, combined repetitive TMS and peripheral coactivation improved tactile discrimination indicated by a lower threshold more than peripheral coactivation alone [6]. Transcranial direct current stimulation (tDCS) is a more recent non-invasive brain stimulation technique for the induction of brain plasticity and thereby modification of sensory, perceptual, motor, cognitive, and behavioral functions [7,8]. Among its many promises, tDCS has been shown to modulate sensory and tactile perception and discrimination. While tDCS, as opposed to TMS, does not induce neuronal action potentials, it is used to modulate the excitability of cortical neurons by hyper- or depolarizing resting-state potentials, thus leading to short- and longer-term brain reorganization and plasticity [9,10,11]. Positively charged stimulation, which causes depolarization of superficial cortical neurons, is named anodal-tDCS (a-tDCS) and negatively charged stimulation, which causes hyperpolarization, is named cathodal-tDCS (c-tDCS) [12]. Sham-tDCS (s-tDCS) is often used to control for the experimental setup itself both in applied [13] and clinical [14] research, but findings are ambivalent: while in some studies s-tDCS does not provide different results from active stimulations, which serves as evidence for a placebo effect [15], in other studies s-tDCS produces different results, refuting a placebo effect [16]. For example, Vaseghi et al. [15] showed in their meta-analysis that c-tDCS of the primary sensory cortex (S1) increased both sensory and pain thresholds in healthy and pathological samples. In other studies, contralateral c-tDCS reduced sensitivity to hot and cold sensations [17], decreased pain perception thresholds [18], and decreased tactile discrimination performance [19]. In a recent study, the tactile discrimination threshold of stroke patients was measured by GOT and it was shown that dual-hemispheric tDCS improved performance in comparison to a sham stimulation group [16] Similarly, low current a-tDCS over S1 of healthy young adults enhanced contralateral index finger discrimination performance in GOT, with no such effects in the s-tDCS condition [13].

Besides the described effects of peripheral or brain stimulation on tactile performance, it has been well established that individual tactile sensitivity and perception at all ages strongly depends on accumulating effects of use or disuse [20,21]. Particularly, extensive daily tactile stimulation as in musicians or blind Braille readers [22,23,24] or in professionals such as surgeons, opticians, or fine mechanics [25,26], may alter tactile perception and related somatosensory cortical representations.

The effects of expertise on stimulation-induced tactile plasticity have also been studied. In 2004, skilled musicians as tactile experts were studied due to evidence that musicians, who practice regularly their sensorimotor skills, have expanded cortical representations and altered grey matter topography in the premotor and primary somatosensory (S1) regions [27,28]. In this study, expert pianists gained more than controls from the tactile stimulation. This significant difference was correlated with the amount of daily practice but not with years of practice [23]. In this study, the finger of interest was directly stimulated by a Hebbian stimulation protocol of tactile coactivation, similar to the one shown to facilitate tactile discrimination performance in healthy participants by Godde and colleagues [3]. A more recent study investigated expertise effects on tactile learning pre- and post-tactile stimulation, using an 8-pinned piezoelectric waver. Significant expertise by intervention interaction was found. The learning effect for the group of experts by occupation was found to be stronger than for the novice controls [25]. The above findings suggest that experts are more susceptible to the effects of tactile stimulation than novices and raise the question of whether tDCS will produce such difference as well.

On the contrary, studies on motor performance hinted at ceiling effects in plasticity in high-performing experts. In one study, bilateral tDCS on young adult professional pianists did not enhance finger dexterity of the pianists, although the age of training-start played a role for some tested features—the pianists who started at an older age improved more with stimulation and practice than pianists who started at a younger age [29]. These results might indicate that optimization of the motor system early in life through extensive training might lead to ceiling effects for later tDCS-induced plasticity. In another study by this group, pianists and untrained participants performed time-sequence finger movements with both hands pre and post bilateral tDCS over primary motor cortex (M1). While the non-musicians improved in both hands from pre to post-performance, the musician group’s performance deteriorated in the ipsilateral digits after c-tDCS and contralateral digits after a-tDCS, suggesting tactile learning ceiling effects due to musical expertise [30].

With the present study, we aimed to investigate the effects of transcranial stimulation and expertise on tactile performance. We examined whether a-tDCS as compared to c- and s-tDCS would facilitate the tactile performance of our participants in two tasks of tactile perception. Based on contradictory findings in the literature on potential ceiling effects in motor experts [29,30], we also investigated if experts in tactile perception, defined as semi-professional musicians who regularly performed on stage in campus events, would gain more from the stimulation than non-musicians. In order to expand our definition of tactile expertise, we later included computer typing as additional activity and quantified tactile expertise as hours of finger-use per week doing computer typing or playing an instrument.

We hypothesized that contralateral a-tDCS would facilitate tactile detection and discrimination by lowering the excitation threshold, and that c-tDCS should have the opposite or no effect. Additionally, s-tDCS should not have any effects on tactile thresholds. Our second hypothesis was that a-tDCS effects would be stronger in tactile experts than in non-experts. Since our expert participant pool consisted of semi-professional musicians, we did not expect ceiling effects in performance improvement.

## 2. Materials and Methods

### 2.1. Participants

Thirty-three participants between 18 and 27 years of age (32 right-handed; 17 females) were recruited from the Jacobs University student population. The left-handed participant was a non-musician with respect to both definitions of expertise and results did not differ when excluding this participant. Thus, we did not exclude him from the analysis. Handedness was formally tested with a modified Edinburgh handedness inventory [31]. All participants signed an informed consent form, were healthy without any neurological disorder and were clean of recreational drug use at least a month before the experiment started. The study was conducted in accordance with local ethics guidelines and the Helsinki convention on studies with human subjects. It was approved by the ethics commission of the German Psychological Society (DGPs; BG 012016). Participants were compensated for their participation with either course credits or monetary compensation.

There were two groups of participants—17 expert musicians (9 females) and 16 non-musicians (8 females). Expert musicians were defined as students that were playing an instrument such as the piano, flute, or violin, and exercising regularly continuously during their studies, resulting in extensive tactile stimulation of the left-hand fingers. The inclusion criterion was that they were proficient enough to play on stage in the university campus events. We applied this definition of the musician group to be able to collect student participants that do not study in a musical school and do not perform music as a profession. The mean amount of years of instrument practice in the musicians group was 9.1 ± 4.7. The non-musicians group did not practice at all musical instruments that require finger tapping (singers and drummers were not disqualified from that group). Additionally, extensive typewriting background was controlled by a self-report of weekly amount of writing [23]. Table 1 presents the number of hours per week musicians and non-musicians reported exercising instrument playing or using their fingers for computer typing. Independent samples t-tests revealed that groups did not differ with respect to computer-typing (*t*(31) = 1.04, *p* = 0.305).

For further analysis, we created another variable called “tactile experience”, defined as the total amount of finger-use (hours per week). This was due to the assumption that tactile expertise maybe expertise-type specific—that is, a specific tactile expertise can be developed by practicing its corresponding tasks, as suggested by Reuter and colleagues [26]. In this case, both musical training and computer-typing would intensely stimulate the fingers and therefore might lead to better tactile perception. The new variable thus was a compound measure composed of both types of expertise together and calculated as the sum of hours per week computer-typing plus hours per week instrument playing.

### 2.2. Experimental Procedure

**Figure 1:**
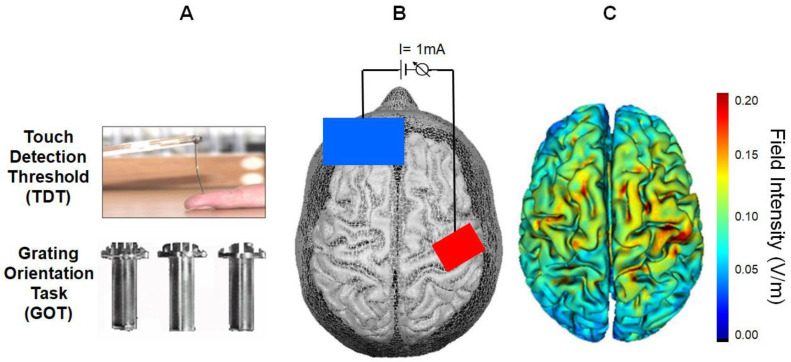
Experimental setup. (**A**) Illustrations of the tactile tests. In the Touch Detection Task (TDT), von Frey filaments were applied vertically to the glabrous finger surface for measuring tactile sensitivity thresholds. In the Grating Orientation Task (GOT), plastic domes with gratings of different widths were applied and participants had to judge the orientation of the gratings. (**B**) For transcranial direct current stimulation (tDCS), the active electrode (red, anode, 24 cm^2^) was placed over the right somatosensory cortex while the large reference electrode (blue, cathode, 96 cm^2^) was placed over the left orbit. (**C**) Field intensity distribution according to computational simulation.

We implemented a sham-controlled, single-blind, cross-over design. Participants were asked to attend three sessions in the psychology lab at Jacobs University Bremen, in intervals of 5–9 days, at similar times of the day. On each day we applied pseudo-randomly a different transcranial direct current stimulation (tDCS) protocol—anodal (a-tDCS), cathodal (c-tDCS), and sham (s-tDCS). Two tactile discrimination tests, TDT and GOT (Figure 1), were given with this order on each day, pre- and post-stimulation. Due to this procedure, we cannot completely exclude order effects. However, because of the short testing duration for TDT we started with this task and did not expect that tDCS effects already vanished when doing the GOT. Tactile tests were conducted on the left little finger (5th digit). Our rationale was that this finger is used by the general population the least, including when computer typing, but it is highly used by musicians, as was suggested by Reuter and colleagues [26]. This also accounts for testing specifically the left hand’s 5th digit, because, in many instruments, such as classical string instruments and guitar, the tapping is performed by the left hand. Additionally, although not very frequently, the 5th digit was already used in similar studies before [29,30]. Duration of each task was 5–10 (TDT) and 10–15 (GOT) min.

#### 2.2.1. Touch Detection Task

TDT was evaluated by applying 12 von-Frey Filaments (Marstocknervtest, Schriesheim, Germany) with descending thickness, which represented the filament’s force from 0.125 to 512 mN on a logarithmic scale. Two-down, one-up procedure was used, and was stopped after six points of return [32]. Threshold was defined as mean of the forces at the six points of return. The log-transformed threshold values were used for further analysis ([33]).

#### 2.2.2. Grating Orientation Task

Tactile spatial discrimination of the left little fingertip (left 5th digit) was evaluated with the GOT by using eight different hemispherical plastic domes with equal width of bars and grooves from 0.25 to 3 mm (width—3, 2, 1.5, 1.2, 1, 0.75, 0.5, 0.25 mm; JVP Domes, Stoelting, Wood Dale, IL, USA). Each dome, starting with the widest and easiest to detect, was implemented in a staircase procedure [33] on the fingertip for twenty times in a horizontal and perpendicular orientation for 1–2 s. The next dome was used only if 75% of the responses were correct using a two-alternative forced-choice procedure. This was repeated until the first dome a participant failed to discriminate 25% of the trials [34,35]. The threshold was calculated using following formula—Equation (1):

Threshold_G75_ = G_low_ + ((0.75 − P_low_)/(P_high_ − P_low_)) × (G_high_ − G_low_) (1)

G = groove width

P = trials correct/number of trials

high = the groove width or probability of correct response on the lowest groove width on

which the subject responded correctly better than 75% of the time.

low = the groove width or probability of correct response on the highest groove width on

which the subject responded correctly less than 75% of the time.

G75 = the hypothetical groove width on which the subject would have scored 75%.

### 2.3. Transcranial Brain Stimulation

TDCS involved continuous administration of weak currents through a pair of surface electrodes attached to the scalp The a-tDCS and c-tDCS paradigms consisted of 15 min of stimulation with 1 mA intensity and 30 s each ramping up and down, and the sham condition consisted of 60 s of anodal stimulation with same intensity just ramping up and down for 30 s. The stimulation was applied by a battery-driven, ramp-controlled DC stimulator (CX-6650, Rolf Schneider Electronics, Gleichen, Germany) and was induced via a NaCl-soaked sponge active electrode (4 × 6 cm; surface area 24 cm^2^) and a reference electrode (8 × 12 cm; surface area 96 cm^2^). The active electrode was placed over the right primary somatosensory cortex (S1) contralateral to the tested finger, whereas the reference electrode was placed on the ipsilateral left orbit [17,36] (Figure 1). We used a 10–20 EEG cap to locate the C4 point and identified the S1 as being located 2 cm posterior to C4 [13,17,34]. Impedance was kept below 10 k ohms. We estimated the resulting electric field intensity in the present study using the HD Explore software (Version 5.0.1; Soterix Medical, New York, NY, USA; [37,38] applying a standard MRI template. According to Nitsche and Paulus [38] a minimum current density of 0.017 mA/cm^2^ is necessary to modify cortical excitability by tDCS in humans. Please note that the current density below the reference electrode did not exceed 0.01 mA/cm^2^ due to the relatively large electrode surface of 96 cm^2^. Current density calculations from our laboratory and previous studies investigating the effects of electrode size on neuromodulation have consistently shown that current densities below 0.017 mA/cm^2^ cannot modulate cortical excitability using tDCS [11,39,40,41]. However, the current density below the active electrode was 0.042 mA/cm^2^ resulting in a maximum field intensity of 0.02 V/m in the right primary somatosensory cortex (S1). During the stimulation period, participants rested relaxed in their chairs without any task. Delay between stimulation and testing was 1–2 min, just the time needed to remove the electrodes and change chair orientation.

### 2.4. Data Analysis

The dataset generated and analyzed during the current study is available in the OSF repository, http://osf.io/r4k9g. Data were analyzed with R [42] using the Jamovi 1.0.7 software environment [43] and RStudio 3.5.1. [42]. General linear model analyses were performed with the GAMLj package for R version 2.0.0 [44].

We conducted two analyses. In analysis 1 we compared the groups of musicians and non-musicians. General linear models (GLM) were calculated separately for TDT and GOT. Thresholds post tDCS stimulation were treated as dependent variables, musical expertise (musician, non-musician) as between factor, and condition (anodal, cathodal, sham) as within factor. Pre tDCS thresholds were included as covariates in the model. As compared to repeated measures ANOVA or ANOVA of change, this procedure should be preferred for controlling baseline differences between groups [45]. Simple effects of tDCS condition or group were estimated for mean level and ± 1 SD of the moderating covariate baseline threshold.

In Analysis 2, similar GLMs were calculated, only the within factor (musical expertise) group was replaced by the covariate tactile experience (Z-transformed). In addition to these two lines of analysis, we assessed the potential effects of musical expertise or tactile experience on baseline TDT and GOT thresholds with one-way ANOVA and correlation analysis, respectively.

Significance threshold was set to α = 0.05.

## 3. Results

Analysis of baseline tactile performance revealed that musicians had lower GOT thresholds at baseline (2.29 ± 0.71 mm, mean and SD) than non-musicians (2.58 ± 0.69 mm; F(1,97) = 4.25, *p* = 0.04). No such effect was revealed for TDT (musicians: −0.835 ± 0.121 mN, non-musicians: −0.827 ± 0.128 mN; F = 0.223, *p* = 0.638). For tactile experience, correlation analysis did not reveal a significant association with baseline TDT or GOT thresholds (Pearson’s R < 0.103, *p* > 0.311).

### 3.1. Analysis 1—Effects of Musical Expertise

**Figure 2:**
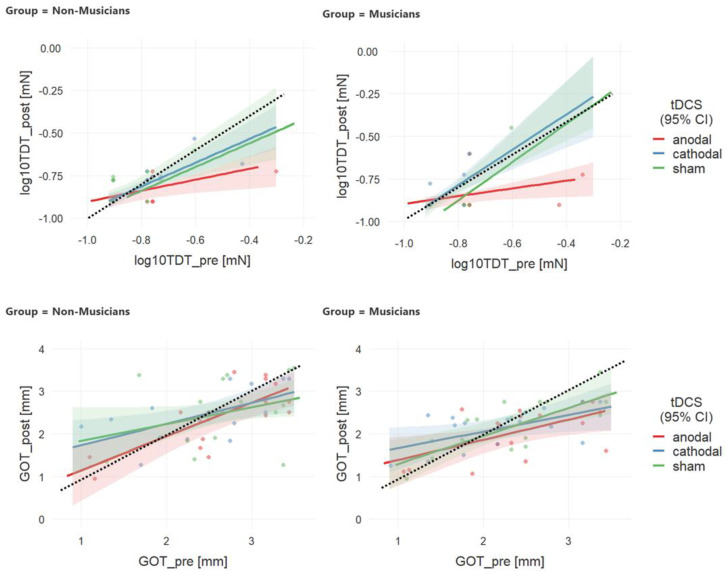
Effects of tDCS and musical expertise on the association between pre- and post-tDCS Table 95. confidence intervals (CI) are shown. The dashed black line is the diagonal indicating no change from pre to post.

Results from analysis 1 are illustrated in Figure 2. For TDT (upper row in Figure 2), relative to pre-tDCS thresholds, post-tDCS thresholds were reduced after anodal stimulation (red line below the diagonal which indicates similar pre and post thresholds). This effect was the stronger the higher the baseline thresholds were. Additionally, the differences between anodal stimulation and the other two stimulation conditions were the largest at higher baseline levels. Regression lines for cathodal and sham stimulation have similar slopes and are close to the diagonal, indicating no change. While the effect of anodal tDCS seems stronger in the groups of musicians, this effect was not significant.

GLM analysis only revealed significant effects of tDCS condition, pre-tDCS thresholds, and condition by pre-tDCS threshold interaction (see Table 2 for detailed statistics). To understand the nature of the moderating effect of baseline thresholds on tDCS condition-effects, simple effects were estimated setting the non-significant factor group in the GLM to zero and averaging across moderating factor levels of mean ± 1 SD. Simple effects estimation confirmed that for mean baseline threshold levels and levels of 1 SD above the mean thresholds after a-tDCS were significantly lower than after c-tDCS (*t* = 2.59, *p* = 0.011 and *t* = 4.71, *p* < 0.001; mean and +1 SD baseline, respectively) or sham tDCS (*t* = 2.72, *p* = 0.008 and *t* = 4.70, *p* < 0.001; Table 3). There was only a non-significant trend for a group by baseline thresholds interaction effect.

For GOT, as illustrated in Figure 2 (lower panel), all regression lines show similar slopes not very different from the diagonal, i.e., post-stimulation thresholds were mostly determined by pre-stimulation thresholds. GLM analysis revealed that post tDCS thresholds were significantly determined by pre tDCS thresholds (see Table 4 for detailed statistics). An only marginally significant effect of musical expertise indicated a trend for lower thresholds in musicians.

### 3.2. Analysis 2—Effects of Tactile Experience

**Figure 3:**
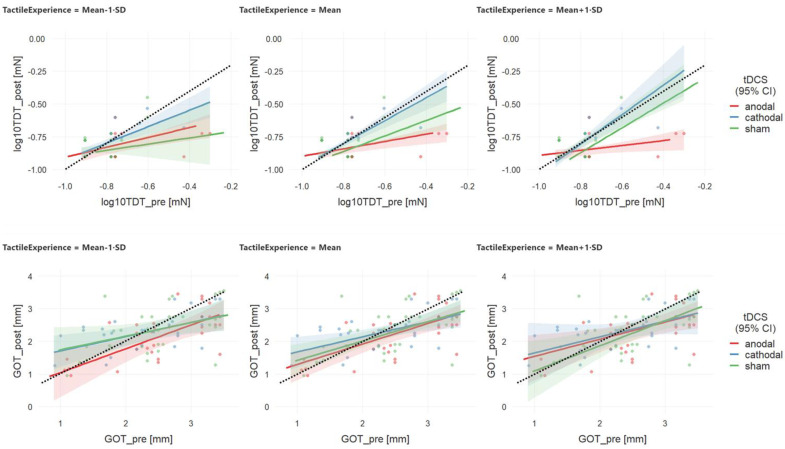
Interaction effects of tDCS condition and baseline threshold levels on TDT threshold changes after stimulation.

For analysis 2, we replaced the between-factor group in the above analysis 1 with the continuous factor tactile experience derived as the combined hours per week computer typing and playing an instrument. As illustrated in Figure 3, again, for TDT (upper panel), but not for GOT (lower panel), higher levels of tactile experience were associated with lower thresholds after a-tDCS as compared to c-tDCS and sham stimulation, particularly at higher baseline levels.

For TDT, GLM analysis confirmed this 3-fold interaction effect of tDCS condition, tactile experience, and baseline threshold (see Table 5 for detailed statistics). Further, the main effects of baseline threshold and tDCS condition, as well as interaction effects of baseline thresholds with condition or tactile experience, were significant. However, those main effects and 2-fold interactions might be ignored given the significant 3-fold interaction effect. Simple effects estimation revealed that thresholds were lower for a-tDCS as compared to c-tDCS across all levels of tactile experience (mean and ±1 SD), particularly at mean and higher (+1 SD) levels of baseline thresholds (Table 6). Thresholds after a-tDCS and sham stimulation differed at mean and high baseline levels and above-average levels of the tactile experience. For GOT, as with analysis 1, GLM only confirmed a significant effect of baseline thresholds on thresholds after tDCS (Table 7).

## 4. Discussion

With the present study, we aimed to investigate the effects of tDCS on tactile detection (TDT) and discrimination (GOT) thresholds dependent on musical and tactile expertise. We confirmed that a-tDCS improved tactile sensitivity in the TDT, while c-tDCS or s-tDCS did not. In analysis 1, TDT thresholds were significantly lower after a-tDCS than after c-tDCS or sham stimulation when baseline thresholds were on mean levels or above. When taking tactile experience into account (Analysis 2), a significant interaction of stimulation condition and experience was again moderated by baseline threshold levels. For a-tDCS, the negative association of tactile experience with TDT thresholds (improved performance) was the stronger the higher the baseline levels were. For GOT, only a significant group effect at baseline was revealed with musicians performing better than non-musicians. However, no effect of tDCS on GOT thresholds was found.

### 4.1. Effects of Different tDCS Conditions

With regards to stimulation conditions, our findings revealed that a-tDCS, but not c-tDCS or sham, improved tactile performance, which is in line with the literature on the effects of anodal stimulation. This effect, however, was specific to TDT and not significant for GOT. This is in contrast to findings by Ragert et al. [13] who reported reduced GOT thresholds after anodal stimulation applied to the primary somatosensory cortex with a similar current density under the anode as in our study. However, from missing significance one cannot conclude on the absence of an effect and different statistics were used in these two studies. While Ragert et al. used repeated-measures ANOVA, we used ANCOVA with baseline performance as covariate which according to Twisk et al. [45] better controls for baseline differences between individuals. Additionally, potential ceiling effects might contribute to reduced anodal stimulation effects in our study. To further elucidate this discrepancy, we performed additional t-tests on pre-to-post threshold differences. Those confirmed significant differences between anodal and cathodal stimulation for TDT (*t*(32) = 2.386, *p* = 0.023) and GOT (*t*(32) = 2.137, *p* = 0.040). For TDT, the pre-to-post threshold differences were also significantly different between anodal and sham stimulation (*t*(32) = 2.571, *p* = 0.015).

The fact that performance improved significantly in one task but not the other may also suggest different neural mechanisms underlying each of the tasks. One could argue that tactile detection is based predominantly on excitability and resulting sensitivity of neurons in the primary somatosensory cortex, whereas spatial discrimination additionally involves higher-order processing steps including secondary and tertiary sensory as well as cognitive areas. Considering that neural mechanisms for tactile detection (TDT) and discrimination (as in the GOT) are highly task-specific, more research is needed to determine the brain pathways that are responsible for each task, although generally, tactile discrimination tasks seem to demand the involvement of higher cortical processing levels than merely the detection of touch [46]

In line with our assumption on the ineffectiveness of c-tDCS in both groups, cathodal stimulation did not significantly improve or impair tactile detection and discrimination. This result persisted in all statistical analyses we conducted. However, again, non-significant findings should not be interpreted in terms of no effects, particularly in small samples.

### 4.2. Expertise Effects on tDCS-Induced Plasticity and Potential Limits for Further Improvement

Against our hypothesis, musicians did not reveal a stronger effect of a-tDCS on tactile thresholds than non-musicians. However, although on visual inspection and taking confidence intervals into account, the interaction effect of tDCS condition and baseline thresholds in the TDT seemed to be based mostly on musicians (Figure 2), this did not reach significance. While it might well be that statistical power was not sufficient to reveal a potential effect of musical expertise in our study, we rather argue that frequent typewriting has a similar effect as regular instrument playing. Hours per week typewriting did not differ between groups and thus group differences might be attenuated by frequent tactile stimulation experiences in the group of non-musicians. This argument well fits with the observation of an association between tactile experience and threshold decline after a-tDCS that was dependent on baseline levels. Further, baseline group differences in TDT thresholds were not significant, i.e., expertise might not have been established sufficiently, to result in learning differences. It cannot be ruled out that using a participant pool that stands in the strict criterion of 10,000 h of musical training or 10 years of musical practice [47], instead of the semi-professional musicians who participated in our study, would have produced different results.

Particularly for GOT, we could not find any moderation of tDCS effects by musical expertise or tactile experience. These findings are opposed to those reported by Reuter and colleagues [25], who revealed that experts gained more than novices from tactile training. It might be that different training paradigms play a role in these deviating results. Reuter et al. (2014) [25] used tactile finger stimulation rather than electrical brain stimulation to induce plasticity.

It might also be that there is a missing link between cortical reorganization as found in specific expert groups like string players [22], pianists [23], or certain occupations [26] and its effect on further plasticity induced by cortical stimulation as reported here. That means, while cortical reorganization related to expertise was described earlier, this possibly did not translate into more improvement by stimulation of the primary somatosensory cortex in experts. A possible explanation could be that expertise was shown to be highly task and expertise-type specific or that the type of stimulation—direct brain stimulation or peripheral stimulation through tactile training —plays a role.

Another support to that idea is that quality of practice [48] and the quantity of daily practice [23,49,50] have been shown to correlate with improvement in performance. However, in the case of musical expertise, we assessed only the level of proficiency in terms of years of playing an instrument and self-reported hours per week, both the average time throughout the years and the average during the last year, before the experiment but did not measure the intensity of practice.

Previous research suggested that for GOT dual-hemisphere tDCS over S1 with anodal electrode on the contralateral and cathodal on the ipsilateral hemisphere improved tactile discrimination in healthy adults significantly more than single hemisphere or sham tDCS [51]. It could also be shown that musicians’ corpus callosum is bigger than that of non-musicians [52] and that there is more interhemispheric functional connectivity in musicians’ than in non-musicians’ brains [53]. Therefore, it might be that the effects of dual-hemispheric tDCS would be significantly different between expertise groups, but currently, we do not have data to support such an assumption.

The interaction analysis with baseline thresholds as covariates revealed for the TDT that in the a-tDCS condition participants with higher baseline thresholds could profit more from anodal stimulation than those with lower thresholds. Probably participants with lower thresholds per se have higher baseline excitability and thus tDCS could have no further effect. On the contrary, in high-threshold participants, enhanced excitability induced by tDCS might act as a gate opener.

A ceiling effect has also been reported by Furuya and colleagues [30]. This would fit with observations by Reuter and colleagues [54] who reported enhanced excitability of somatosensory cortex, as revealed by enlarged N70 event-related potential amplitudes, in experts as compared to novices. Additional anodal (facilitating) stimulation might thus be less effective in experts as compared to novices. However, a general limit for further improvement in experts by a-tDCS was not found, i.e., we did not observe a minimum threshold from which experts (or non-experts) could not further improve from. Findings from c-tDCS and s-tDCS also suggest that there was no general ceiling effect. A possible explanation for differences in results between Furuya and colleagues [30] and Ragert and colleagues [23] who, similarly to us, did not find limits in further training, is the type of stimulation: while the first used t-DCS on bilateral S1, the latter directly stimulated the fingers to co-activate different receptive fields and thereby facilitate tactile performance.

### 4.3. Limitations

The musical expert participant sample entailed only semiprofessional musicians and we found typical inter-individual differences both in performance before stimulation and in reaction to stimulation even within the expertise groups. Additionally, our musician group’s average instrument play in the last year before the experiment was lower than earlier in their lives, most probably due to their academic demands and not having their own instrument with them in the university campus. These issues strengthen our argument on the need to define experts more rigorously both in terms of their skills and in terms of the expertise-type specificity of the task experimentally performed.

When we defined expertise as a summation of both musical and computer-typing experience (Analysis 2), we did not find significant main effects of expertise. Not much has been studied to date regarding the effects of typewriting as expertise, but it is worthwhile to note that Reuter and colleagues [26] found that typewriting had an effect approaching significance on tactile perception.

This study has several other limitations. First, blinding of the participants was not tested formally. Additionally, this was a single-blind study and the experimenter’s knowledge of the condition at each time may have caused bias in testing [55]. Second, the experimenter tested the participants manually. It might be that a semi-automatic device, as exists for GOT [13], might have compensated for the study not being double-blinded. Third, possible ceiling-effects cannot be excluded but results from the interaction analysis with baseline performance levels as covariate suggest that this was not the case. Another limitation is that the age of initiation of musical education was not considered in our study, but was suggested in previous literature [22,29].

Further, despite significance, effect sizes are rather small. Individual differences in head shape and tissue composition might have lowered electrical field strength in some participants to ineffective levels and contributed to non-explained variance. Future studies might use higher tDCS amplitudes or different electrode layouts to overcome this limitation.

Finally, potential order effects in testing (TDT first, GOT second) could potentially contribute to fewer effects on GOT.

In future studies, it is recommended to use a defined protocol, agreed by different authors of the field, with the same stimulation time and intensity, and to identify reasons for interindividual differences in response to tDCS. This problem was brought forward several times before, including by Trembley and colleagues [56], who experimented on differences in outcomes after different anodal stimulation periods (10 or 20 min) and different stimulation intensities (1 or 2 mA)—but found no significant results. Such a protocol will allow for a comparison between experiments and a more comprehensive meta-analysis that will optimize future research. Furthermore, as mentioned earlier, it is recommended to collect expert musicians who play only one type of instrument to be able to draw more decisive conclusions.

## 5. Conclusions

In summary, only anodal tDCS but not cathodal or sham stimulation was associated with improved tactile performance in our study. When looking into expertise, no significant main effects were shown—being it musical expertise, computer-typing expertise, or a combination of these two. Nevertheless, a potential gating effect of tactile expertise on a-tDCS induced plasticity or excitability changes could be revealed for low performing participants.

## Figures and Tables

**Table 1:** Descriptive statistics. Mean values (and SD) of amount of finger use in instrument playing and computer-typing per musical expertise group. HpW: Hours per Week.

Musical Expertise Group [N]	Age [Years]	Instrument Playing [HpW] 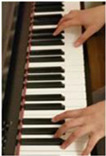	Computer-Typing [HpW] 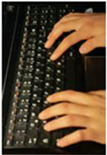
Musicians: 17	19.8 (2.1)	6.6 (5)	9.6 (9.9)
Non-musicians: 16	21.1 (1.5)	-	13 (8.4)

**Table 2:** General linear model (GLM) results for TDT with musical expertise (experts, novices) as between factor; *: significant group differences, *p* < 0.05. Significant effects in bold.

	SS	df	F	*p*	η^2^p
**Model**	**0.5359**	**11**	**11.50**	**<0.001**	**0.592**
**log10TDT_pre [mN]**	**0.1999**	**1**	**47.17**	<0.001 *	**0.352**
**tDCS**	**0.0338**	**2**	**3.99**	0.022 *	**0.084**
Group	0.0116	1	2.73	0.102	0.030
**tDCS × log10TDT_pre [mN]**	**0.0264**	**2**	**3.11**	0.049 *	**0.067**
tDCS × Group	0.0176	2	2.07	0.132	0.045
Group × log10TDT_pre [mN]	0.0125	1	2.94	0.090	0.033
tDCS × Group × log10TDT_pre [mN]	0.0181	2	2.14	0.124	0.047
Residuals	0.3686	87			
Total	0.9045	98			

**Table 3:** Simple effects estimates of tDCS condition at fixed levels of baseline thresholds for TDT; *: significant group differences, *p* < 0.05. Significant effects in bold.

Moderator Levels				95% Confidence Interval				
log10TDT_pre [mN]	Contrast	Estimate	SE	Lower	Upper	df	t	*p*
Mean − 1·SD	Cathodal-anodal	−0.0309	0.0235	−0.07758	0.0159	87.0	−1.31	0.193
	Sham-anodal	−0.0341	0.0236	−0.08109	0.0128	87.0	−1.44	0.152
**Mean**	**Cathodal-anodal**	**0.0428**	**0.0165**	**0.00991**	**0.0756**	**87.0**	2.59 *	**0.011**
	**Sham-anodal**	**0.0447**	**0.0164**	**0.01199**	**0.0773**	**87.0**	2.72 *	**0.008**
**Mean + 1·SD**	**Cathodal-anodal**	**0.1164**	**0.0247**	**0.06732**	**0.1655**	**87.0**	4.71 *	**<0.001**
	**Sham-anodal**	**0.1235**	**0.0263**	**0.07125**	**0.1757**	**87.0**	4.70 *	**<0.001**

**Table 4:** GLM results for GOT with musical expertise (experts, novices) as between factor; *: significant group differences, *p* < 0.05. Significant effects in bold.

	SS	df	F	*p*	η^2^p
**Model**	**17.5703**	**11**	**5.8345**	**<0.001**	**0.425**
tDCS	0.9336	2	1.7050	0.188	0.038
Group	0.8412	1	3.0728	0.083	0.034
**GOT_pre [mm]**	**12.9271**	**1**	**47.2188**	<0.001 *	**0.352**
tDCS × Group	0.0238	2	0.0434	0.958	0.001
Group × GOT_pre [mm]	0.0359	1	0.1311	0.718	0.002
tDCS × GOT_pre [mm]	0.2867	2	0.5237	0.594	0.012
Group × tDCS × GOT_pre [mm]	0.6322	2	1.1546	0.320	0.026
Residuals	23.8179	87			
Total	41.3882	98			

**Table 5:** GLM results for TDT with musical expertise (experts, novices) as between factor; *: significant group differences, *p* < 0.05. Significant effects in bold.

	SS	df	F	*p*	η^2^p
**Model**	**0.59394**	**11**	**15.126**	**<0.001**	**0.657**
**log10TDT_pre [mN]**	**0.28749**	**1**	**80.535**	<0.001 *	**0.481**
TactileExperience	0.00135	1	0.379	0.540	0.004
**tDCS**	**0.02546**	**2**	**3.566**	0.032 *	**0.076**
tDCS × TactileExperience	0.00340	2	0.476	0.623	0.011
**tDCS × log10TDT_pre [mN]**	**0.08393**	**2**	**11.756**	<0.001 *	**0.213**
**TactileExperience × log10TDT_pre [mN]**	**0.03955**	**1**	**11.078**	0.001 *	**0.113**
**tDCS × TactileExperience × log10TDT_pre [mN]**	**0.07585**	**2**	**10.624**	<0.001 *	**0.196**
Residuals	0.31057	87			
Total	0.90451	98			

**Table 6:** Simple effects estimates of tDCS condition at fixed levels of baseline thresholds for TDT; *: significant group differences, *p* < 0.05. Significant effects in bold.

Moderator Levels					95% Confidence Interval				
Tactile Experience	log10TDT _pre [mN]	Contrast	Estimate	SE	Lower	Upper	df	t	*p*
Mean − 1 SD	Mean − 1 SD	Cathodal-anodal	0.01735	0.0293	−0.04086	0.07555	87.0	0.592	0.555
		sham-anodal	0.04011	0.0330	−0.02555	0.10576	87.0	1.214	0.228
	**Mean**	**Cathodal-anodal**	**0.04847**	**0.0213**	**0.00603**	**0.09090**	**87.0**	2.270 *	**0.026**
		sham-anodal	0.02278	0.0222	−0.02136	0.06693	87.0	1.026	0.308
	**Mean + 1 SD**	**Cathodal-anodal**	**0.07959**	**0.0258**	**0.02828**	**0.13089**	**87.0**	3.083 *	**0.003**
		sham-anodal	0.00546	0.0397	−0.07349	0.08441	87.0	0.138	0.891
**Mean**	**Mean − 1 SD**	**Cathodal-anodal**	**−0.03489**	**0.0216**	**−0.07790**	**0.00813**	**87.0**	−1.612 *	**0.111**
		sham-anodal	−0.01078	0.0227	−0.05600	0.03444	87.0	−0.474	0.637
	**Mean**	**Cathodal-anodal**	**0.03883**	**0.0149**	**0.00922**	**0.06845**	**87.0**	2.606 *	**0.011**
		sham-anodal	0.02768	0.0157	−0.00347	0.05883	87.0	1.766	0.081
	**Mean + 1 SD**	**Cathodal-anodal**	**0.11256**	**0.0213**	**0.07022**	**0.15489**	**87.0**	5.285 *	**<0.001**
		**sham-anodal**	**0.06614**	**0.0283**	**0.00993**	**0.12235**	**87.0**	2.339 *	**0.022**
**Mean + 1 SD**	**Mean − 1 SD**	**Cathodal-anodal**	**−0.08712**	**0.0346**	**−0.15582**	**−0.01843**	**87.0**	−2.521 *	**0.014**
		sham-anodal	−0.06167	0.0320	−0.12521	0.00187	87.0	−1.929	0.057
	Mean	Cathodal-anodal	0.02920	0.0220	−0.01455	0.07296	87.0	1.327	0.188
		sham-anodal	0.03258	0.0226	−0.01240	0.07755	87.0	1.440	0.154
	**Mean + 1 SD**	**Cathodal-anodal**	**0.14553**	**0.0314**	**0.08312**	**0.20794**	**87.0**	4.635 *	**<0.001**
		**sham-anodal**	**0.12682**	**0.0267**	**0.07367**	**0.17997**	**87.0**	4.742 *	**<0.001**

**Table 7:** GLM results for GOT with musical expertise (experts, novices) as between factor; *: significant group differences, *p* < 0.05. Significant effects in bold.

	SS	df	F	*p*	η^2^p
**Model**	**16.5792**	**11**	**5.2854**	**<0.001**	**0.401**
TactileExperience	0.0270	1	0.0946	0.759	0.001
tDCS	0.7019	2	1.2308	0.297	0.028
**GOT_pre [mm]**	**13.6236**	**1**	**47.7752**	<0.001 *	**0.354**
tDCS × TactileExperience	0.3849	2	0.6748	0.512	0.015
GOT_pre [mm] × TactileExperience	0.0241	1	0.0845	0.772	0.001
GOT_pre [mm] × tDCS	0.2279	2	0.3996	0.672	0.009
GOT_pre [mm] × TactileExperience × tDCS	0.4126	2	0.7235	0.488	0.016
Residuals	24.8090	87			
Total	41.3882	98			
